# High-resolution Diffusion-weighted Imaging to Detect Changes in Tumor Size and ADC, and Predict Adverse Biopsy Histology during Prostate Cancer Active Surveillance

**DOI:** 10.1158/2767-9764.CRC-24-0009

**Published:** 2024-03-27

**Authors:** Rola Saouaf, Yibin Xie, Sungjin Kim, Yaniv Raphael, Christopher Nguyen, Daniel Luthringer, Timothy J. Daskivich, Eric Lo, Mourad Tighiouart, Debiao Li, Hyung L. Kim

**Affiliations:** 1 Department of Imaging, Cedars Sinai Medical Center, Los Angeles, California.; 2 Biomedical Imaging Research Institute, Cedars Sinai Medical Center, Los Angeles, California.; 3 Cedars Sinai Medical Center, Biostatistics and Bioinformatics Research Center, Los Angeles, California.; 4 Cardiovascular Innovation Research Center, Heart Vascular Thoracic Institute, Cleveland Clinic, Cleveland, Ohio.; 5 Department of Pathology Cedars Sinai Medical Center, Los Angeles, California.; 6 Department of Urology, Cedars Sinai Medical Center, Los Angels, California.

## Abstract

**Purpose:**

Majority of men with low-risk prostate cancer can be managed with active surveillance (AS). This study evaluates a high-resolution diffusion-weighted imaging (HR-DWI) technique to predict adverse biopsy histology (AH), defined as Gleason score ≥7 on any biopsy or ≥3 increase in number of positive biopsy cores on systematic biopsies. We test the hypothesis that high-grade disease and progressing disease undergo subtle changes during even short intervals that can be detected by HR-DWI.

**Experimental Design:**

In a prospective clinical trial, serial multiparametric MRIs, incorporating HR-DWI and standard DWI (S-DWI) were performed approximately 12 months apart prior to prostate biopsy (*n* = 59). HR-DWI, which uses reduced field-of-view and motion compensation techniques, was compared with S-DWI.

**Results:**

HR-DWI had a 3-fold improvement in spacial resolution compared with S-DWI as confirmed using imaging phantoms. For detecting AH, multiparametric MRI using HR-DWI had a sensitivity of 75% and specificity of 83.9%, and MRI using S-DWI had a sensitivity of 71.4% and specificity of 54.8%. The AUC for HR-DWI was significantly higher (0.794 vs. 0.631, *P* = 0.014). Secondary analyses of univariable predictors of AH showed tumor size increase [OR 16.8; 95% confidence interval (CI): 4.06–69.48; *P* < 0.001] and apparent diffusion coefficient (ADC) decrease (OR 5.06; 95% CI: 1.39–18.38; *P* = 0.014) on HR-DWI were significant predictors of AH.

**Conclusion:**

HR-DWI outperforms S-DWI in predicting AH. Patient with AH have tumors that change in size and ADC that could be detected using HR-DWI. Future studies with longer follow-up should assess HR-DWI for predicting disease progression during AS.

**Significance:**

We report on a prospective clinical trial using a MRI that has three times the resolution of standard MRI. During AS for prostate cancer, two high-resolution MRIs performed approximately a year apart can detect tumor changes that predict the presence of aggressive cancers that should be considered for curative therapy such as prostatectomy or radiation.

## Introduction

Prostate cancer is the most frequently diagnosed cancer in men and the second most common cause of cancer-related death in U.S. men (1). However, the vast majority of men diagnosed with localized prostate cancer do not die of their disease, even if curative therapy is not administered (2, 3). Furthermore, definitive therapies for prostate cancer can negatively impact quality-of-life by producing side effects such as permanent urinary incontinence (7%–14% posttreatment) and erectile dysfunction (44%–51% posttreatment; ref. 4). Therefore, the National Comprehensive Cancer Network (NCCN) guidelines recommends active surveillance (AS) for men with low-risk and for even some men with intermediate-risk prostate cancer, which together represents the majority of newly diagnosed prostate cancers (5). A barrier to AS is that clinical tools for risk-stratifying and monitoring men with newly diagnosed prostate cancer have limited accuracy and concerns about underestimating cancer risk lead to overtreatment. Therefore, better diagnostic tools are clearly needed.

Multiparametric MRI, including diffusion-weighted imaging (DWI), is the gold-standard for detecting and localizing prostate cancer. DWI is sensitive to the diffusion of water molecules interacting with surrounding macromolecules. The latest version of the Prostate Imaging-Reporting and Data System (PI-RADS) relies primarily on DWI to identify tumors in the peripheral zone, which is where most prostate cancers form. However, partly due to limited spatial resolution, standard DWI (S-DWI) has difficulty detecting small tumors (6) or small changes in tumor size (7), despite attempts to standardize the reporting of serial MRI findings (8).

We have previously shown that higher resolution diffusion-weighted imaging (HR-DWI) improves detection of small tumors (9, 10). For this study, we developed a high-resolution DWI sequence (HR-DWI) with approximately 3-fold improvement in the spatial resolution of S-DWI, without excessively extending scan time. This was achieved by limiting the field-of-view and focusing on the prostate (11) such that peripheral signals that can produce imaging artifacts were suppressed. Spatial resolution was also improved by reducing effects of prostate motion that can result from involuntary muscle contractions or rectal peristalsis, which is particularly problematic when detecting microscopic movement of water. To reduce signal loss from prostate motion, the timing, strength, and sequences of the magnet field were optimized (12).

**FIGURE 1:**
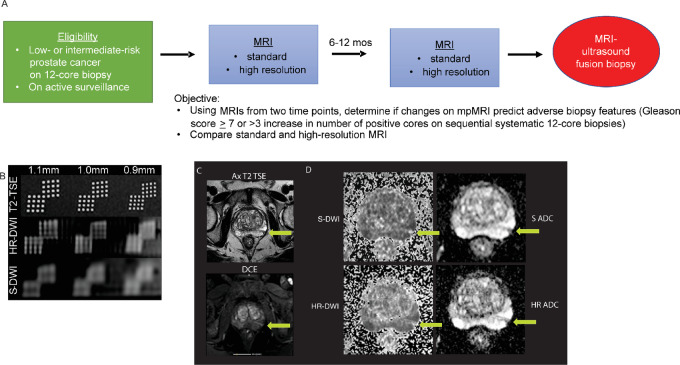
Clinical trial schema to compare HR-DWI with S-DWI. **A,** Prospective clinical trial enrolled men on prostate cancer AS. **B,** T2-TSE image, HR-DWI, and S-DWI of a standard spatial resolution American College of Radiology phantom. The T2-TSE image was acquired at higher resolution (0.6 × 0.6 mm) for visual comparison. The HR-DWI (1.1 × 1.1 mm) and S-DWI (1.6 × 2.0 mm) were acquired with *b* value = 800 s/mm^2^. **C** and **D,** Representative MRI for an AS prostate cancer patient. **C,** Linear lesion seen on T2-TSE and DCE images are highlighted with arrows. **D,** DWI and ADC for S-DWIs and HR-DWIs. TSE, turbo spin echo; HR-DWI, high-resolution diffusion-weighted image; S-DWI, standard diffusion-weighted image; DCE, dynamic contrast enhanced; ADC, apparent diffusion coefficient.

We prospectively evaluated the clinical impact of our HR-DWI in a cohort of 59 men with prostate cancer who were undergoing AS (Fig. 1A). It has been reported that S-DWI cannot accurately determine tumor size (13), and serial biopsies are performed during AS to assess tumor grade and estimate tumor burden. These biopsies are uncomfortable, and come with significant risks, such as a 4% hospitalization rate due to complications that include sepsis and rectal bleeding when performed transrectally (14). Better imaging may identify multifocal tumors, serially assess tumor size, and predict tumor grade, which may lead to enhanced risk stratification and fewer biopsies during AS. In a prospective clinical trial, men under AS were imaged at baseline and approximately 12 months later, using both HR-DWI and S-DWI, before undergoing a biopsy. We hypothesized that HR-DWI, but not S-DWI, will detect subtle changes in tumor size or apparent diffusion coefficient (ADC) that can predict adverse biopsy histology (Gleason score 7–10 or ≥3 increase in the number of positive cores). The criteria for adverse biopsy histology (AH) was intended to capture patients with high-grade component (i.e., Gleason grade 4–5) in their cancer and patients with progressing disease because these patients may need more intense surveillance or even definitive local therapy.

## Materials and Methods

### Patients

A prospective clinical trial (Pro00049177; ref. 15) was approved by the Institutional Review Board in accordance with the Declaration of Helsinki and registered with ClinicalTrials.gov (NCT03292874). Subjects were enrolled between September 2017 and December 2022. The primary inclusion criterion was men on prostate cancer AS with no prior treatment for prostate cancer. Participants were enrolled after providing written informed consent and received two MRI scans approximately 12 months apart (Fig. 1A). Clinical MRI reports using PI-RADS were generated by one of two genitourinary (GU) radiologists (R. Saouaf and Y. Raphael) using all available images, including S-DWI and HR-DWI. The radiologist had access to all clinical information, including any prior biopsies. This increased the likelihood of sampling any suspicious lesions. PI-RADS 3–5 lesions were biopsied using the UroNav system (Philips). Patients underwent an MRI-ultrasound fusion biopsy (two cores per lesion seen on second MRI) along with a standard 12-core systematic biopsy. Enrollment was paused between 2020 and 2022 because of the COVID pandemic and 5 patients that obtained the first MRI could not return for their second MRI and were replaced.

### MRI Scanning Protocol

All study participants underwent imaging on a 3.0T clinical MRI system (MAGNETOM Biograph mMR, Siemens Healthineers) equipped with a 12-channel phased array body matrix coil (Supplementary Table S1). No endorectal coil was employed. Sequential imaging was conducted for S-DWI and HR-DWI following the standard guidelines for pelvic localization. In addition, anatomic scans incorporated both T1- and T2-weighted turbo spin echo techniques. The improved spatial resolution of HR-DWI was made possible by reduced imaging volume via suppressing signals from the surrounding tissues (11, 16). In addition, signal loss due to motion during the scan was decreased by a motion-compensating gradient design (12). Dynamic contrast-enhanced (DCE) MRI was acquired after DWI, which consisted of a prescan, a series of continuous acquisitions of 28 volumes during contrast delivery, and a final 9-minute delay postscan.

### Biopsy and Histologic Examination

All patients underwent ultrasound-guided transrectal prostate biopsy using a side-fire biopsy probe (BK Medical Flex Focus 400) by one of two urologists with a urologic oncology-specific clinical practice (H.L. Kim and T.J. Daskivich). All biopsies were reviewed by GU pathologists with expertise in prostate cancer who were blinded to MRI findings.

### Image Analysis

After the last enrolled patient had their study biopsy, all images were re-read to test the study hypothesis. Suspicious prostatic lesions were identified and scored according to PI-RADS, v2.1 (17) by consensus of two GU radiologists (R. Saouaf: 25 years, Y. Raphael: 5 years of experience in prostate cancer MRI characterization) who were blinded to all pathology results and type of DWI protocol (HR vs. S). The S-MRI (multiparametric MRI with S-DWI) and HR-MRI (multiparametric MRI with HR-DWI) were read in batches, at least 3 weeks apart to decrease chance of recall and bias. Within each batch, the images were randomized. For image analysis, a calculated *b* value of 2,000 s/mm^2^ was used and regions of interest were drawn on ADC images with three-dimensional volume and average ADC generated by DynaCad software (Phillips). Following consensus review by the radiologists, tumor size (determined from multislide planimetry) and ADC determined by each radiologist for each tumor were averaged. MRI findings were considered positive for disease progression if any tumor increased by >0.2 cm^3^ or ADC decreased by more than 20. To decrease risk of overfitting, these cutoffs were selected by our radiologists prior to data analysis.

### Statistical Analysis

Study design and data collection were planned prior to study initiation. If HR-MRI but not S-MRI can detect a change in tumor size or ADC, the study had at least 80% power to detect a mean change in tumor volume of 0.18 cm^3^ and a 50% decrease in ADC between patients with or without AH on biopsy. The biopsy was considered the gold-standard reference for comparing imaging methods in a per-patient analysis.

The primary endpoint was AH defined as either a Gleason score of 7 or more on any biopsy, or an increase of 3 or more positive cores on systematic biopsies. Areas under the ROC curve (AUC) were compared between models with standard and high-resolution MRI variables using the nonparametric method described by DeLong (18). All analyses were performed using R package version 4.0.5 (19) with two-sided tests at a significant level of 0.05.

### Data Availability

The data generated in this study are not publicly available because the information may compromise patient privacy but are available upon reasonable request from the corresponding author.

## Results

### HR-DWI

PI-RADS relies on DWI to identify and characterize most prostate tumors. Enhanced spatial resolution for our HR-DWI was verified using standard American College of Radiology phantoms (20). In Fig. 1B, the T2-weighted images are provided for visual comparison. HR-DWI was able to resolve the individual holes spaced 1 mm apart but not 0.9 mm apart, indicating a resolution between 0.9 × 0.9 mm and 1.0 × 1.0 mm. S-DWI was not able to resolve any of the grids in the figure. The resolution of S-DWI used in our routine clinical setting is 1.6 × 2.0 mm. Therefore, HR-DWI had approximately a 3-fold improvement in resolution when compared with the acquired resolution of S-DWI. Figure 1C and D show an example of S-MRI and HR-MRI where the linear shape of the lesion seen on T2 and DCE images is better outline by HR-DWI when compared with S-DWI.

### Clinical Trial

In a prospective clinical trial (Fig. 1A), we investigated whether HR-DWI, with it improved resolution over S-DWI, can detect changes in prostate tumor size or ADC that can in turn predict the presence of clinically significant cancer (CSC). There are no universally accepted criteria for CSC. However, the NCCN provides widely accepted definitions for CSC (5). We selected, from these definitions, the criteria that can be assessed using biopsies. We defined AH as either overall Gleason score of 7 or more on any biopsy, or an increase of 3 or more positive cores on serial systematic biopsies. Both criteria, as noted by the NCCN, may necessitate definitive local treatments such as radical prostatectomy or radiotherapy. Thus, the ability to predict AH noninvasively, using serial MRIs, may be clinically valuable. Moreover, a positive clinical trial would confirm the clinical importance of the enhanced resolution provided by HR-DWI.

Patient demographics are summarized in Table 1. The patients were representative of a national prostate cancer cohort (ref. 21; Supplementary Table S2). Patients had NCCN low- or intermediate-risk prostate cancer and were on AS. There was one patient where the treating physician elected to repeat the MRI and perform biopsy 6 months after the first study MRI because of an increase in PSA. AH was detected in 30 patients. In these patients, 25 (42%) had Gleason score ≥7, 5 (8%) had progression in number of positive cores, and 2 (3%) had both. No patient had a study biopsy showing Gleason score 4+3 or higher. The average number of years between first prostate cancer diagnosis and the first study MRI was 1.5 years (range: 0.1–8.0 years).

**TABLE 1:** Baseline demographics

Age (years)	65 yo (6.7, 47–79)
Race	
Caucasian	44 (75)
Asian	7 (12)
African American	6 (10)
Hispanic	2 (3)
PSA	6.0 (2.8, 0.7–18.8)
Gleason score	
3 + 3	45 (76)
3 + 4	14 (24)
No. of positive systemic cores	2.9 (2.4, 0–8)
No. of prior biopsies	
0	41 (69)
1	8 (14)
>1	10 (17)
Stage	
T1c	58 (98)
T2a	1 (2)
Months between MRIs	10.9 (2.2, 6–19)

NOTE: Data are presented as number of patients (%) or mean (SD, range).

Abbreviation: yo, years old.

### Primary Clinical Outcome

After the last enrolled patient had their study biopsy, all images were re-read to test the study hypothesis. Suspicious prostatic lesions were identified and scored according to PI-RADS by consensus of two GU radiologists who were blinded to all pathology results and type of DWI protocol. The S-MRI (multiparametric MRI with S-DWI) and HR-MRI (multiparametric MRI with HR-DWI) were read in batches, to decrease the risk of one DWI protocol influencing the interpretation of the other DWI protocol for an individual patient.

The primary endpoint of the clinical trial was the presence of AH on prostate biopsy. The primary hypothesis was that change in tumor size or ADC as detected by HR-MRI would predict AH. The presence of AH was determined at the patient level because clinical decisions, such as the decision to transition from AS to definitive local therapy, are made at the patient level. Furthermore, it is not possible to consider imaging findings and histology at the tumor level when prostatectomies are not performed, and in contemporary practice most low and intermediate grade tumors are managed with AS and not surgery.

**FIGURE 2:**
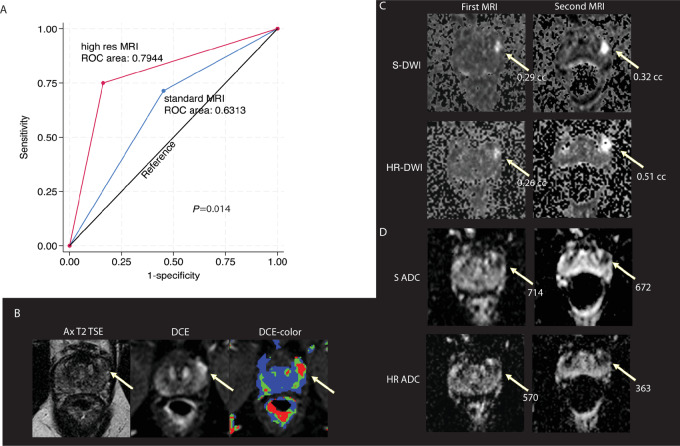
Comparing high-resolution MRI and standard MRI. **A,** Multiparametric prostate MRIs incorporating either S-DWI or HR-DWI were compared. Patients on prostate cancer As were enrolled in a clinical trial and changes in tumor size or ADC as seen on MRI studies approximately 12 months apart were used to predict adverse biopsy histology. **B**–**D,** Representative MRIs taken 12 months apart for an AS patient enrolled in the clinical trial. **B,** T2-TSE, DCE, and DCE (color) images from the second MRI are shown and a prostate lesion is highlighted with arrows. **C,** S-DWI and HR-DWI images from both timepoints are shown. **D,** Standard and high-resolution ADC maps from both timepoints are shown. DWI, diffusion-weighted image; ADC, apparent diffusion coefficient; TSE, turbo spin echo; DCE, dynamic contrast enhanced.

The study was highly significant for the primary outcome (*P* < 0.001) with OR of 15.6 [95% confidence interval (CI): 4.32–56.31] for HR-MRI predicting AH. S-MRI was a much weaker predictor of AH (*P* = 0.044) with an OR of 3.04 (95% CI: 1.03–8.96). As shown in Table 2, although both HR-MRI and S-MRI were significant predictors of AH, HR-MRI had greater sensitivity (75% vs. 71%) and specificity (84% vs. 54.8%). The negative predictive value of HR-MRI and S-MRI were 78.8% and 68%, respectively. If patients with imaging that was negative for progression avoided biopsies, 33/59 (56%) of patients having HR-MRI and 25/59 (42%) of patient having S-MRI would have avoided a biopsy. As illustrated in Fig. 2A, the AUC for HR-MRI was significantly higher than the AUC for S-MRI (0.794 vs. 0.631; *P* = 0.014). Figure 2B–D shows an example of a case with AH where the HR-DWI and corresponding ADC map show a greater change in lesion size and ADC, respectively, than S-DWI. The axial T2 TSE and DCE images are shown for reference (Fig. 2B). This tumor is not well visualized on T2 but clearly visualized on DCE images. On the second MRIs, the lesion measures larger on HR-DWI than S-DWI (Fig. 2C). On the second MRI, the ADC of the lesion is lower on HR-DWI than S-DWI (Fig. 2D). Biopsy showed Gleason score 7, suggesting that HR-MRI is better than S-MRI at detecting imaging changes associated with tumor growth and progression.

**TABLE 2:** Size progression or ADC progression^a^ as predictor of adverse biopsy histology^b^

	Standard MRI	High-resolution MRI
Sensitivity	71.4%	75.0%
Specificity	54.8%	83.9%
Accuracy	62.7%	79.7%
AUC	0.631	0.794

^a^>0.2cc increase in volume of at least one PI-RADS 3–5 lesion or >20 decrease in ADC of at least one PI-RADS 3–5 lesion.

^b^≥3 increase in number of positive cores on sequential systematic 12-core biopsies or Gleason score ≥7 on biopsy.

### Secondary Clinical Outcomes

Several MRI findings were considered as univariate predictors of AH. Table 3 summarizes the prediction of AH using the S-MRI. Change in tumor size was the only significant predictor of AH (OR 6.17; 95% CI: 1.97–19.35; *P* = 0.002). Table 4 summarizes the prediction of AH using the HR-MRI. Increase in tumor was highly predictive of AH (OR 16.8; 95% CI: 4.06–69.48; *P* < 0.001). Decrease in ADC was also predictive of AH (OR 5.06; 95% CI: 1.39–18.38; *P* = 0.014). Although an increase in PI-RADS score for any lesion (*P* = 0.097) and presence of new PI-RADS 3–5 lesion on the second HR-MRI (*P* = 0.052) were not significant predictors of AH, both predictors approached statistical significance.

**TABLE 3:** Standard MRI

	Adverse biopsy histology^a^			
	No	Yes	OR (95% CI)	*P*-value
Size progression^b^				
No	24 (71)	10 (29)	1 (Reference)	0.002
Yes	7 (28)	18 (72)	6.17 (1.97–19.35)	
ADC progression^c^				
No	22 (58)	16 (42)	1 (Reference)	0.270
Yes	9 (43)	12 (57)	1.83 (0.62–5.39)	
PI-RADS progression^d^				
No	29 (57)	22 (43)	1 (Reference)	0.112
Yes	2 (25)	6 (75)	3.95 (0.73–21.51)	
New lesion (PI-RADS 3–5)				
No	18 (51)	17 (49)	1 (Reference)	0.836
Yes	13 (54)	11 (46)	0.90 (0.32–2.54)	

^a^Data are presented as number of patients (row %).

^b^>0.2cc increase in volume of at least one PI-RADS 3–5 lesion.

^c^>20 decrease in ADC of at least one PI-RADS 3–5 lesion.

^d^Increase in PI-RADS of at least one PI-RADS 3–5 lesion.

**TABLE 4:** High-resolution MRI

	Adverse biopsy histology^a^			
	No	Yes	OR (95% CI)	*P*-value
Size progression^b^				
No	28 (74)	10 (26)	1 (Reference)	<0.001
Yes	3 (14)	18 (86)	16.80 (4.06–69.48)	
ADC progression^c^				
No	27 (63)	16 (37)	1 (Reference)	0.014
Yes	4 (25)	12 (75)	5.06 (1.39–18.38)	
PI-RADS progression^d^				
No	30 (57)	23 (43)	1 (Reference)	0.097
Yes	1 (17)	5 (83)	6.52 (0.71–59.71)	
New lesion (PI-RADS 3–5)				
No	12 (40)	18 (60)	1 (Reference)	0.052
Yes	19 (66)	10 (34)	0.35 (0.12–1.01)	

^a^Data are presented as number of patients (row %).

^b^>0.2cc increase in volume of at least one PI-RADS 3–5 lesion.

^c^>20 decrease in ADC of at least one PI-RADS 3–5 lesion.

^d^Increase in PI-RADS of at least one PI-RADS 3–5 lesion.

## Discussion

Our study shows that HR-DWI can predict AH in men undergoing AS for prostate cancer more accurately than S-DWI. HR-DWI was able to detect small changes in tumor size and ADC that were not detectable by S-DWI, and these changes were significantly associated with AH. In a prospective clinical trial, we showed that HR-DWI was able to accurately predict the presence of adverse biopsy histology in 80% of cases, while S-DWI only achieved an accuracy of 63%. These findings suggest that HR-DWI could be valuable in monitoring men on prostate cancer AS, potentially reducing the lifetime number of surveillance biopsies. This would not only improve patient safety and quality-of-life, but also reduce the burden of prostate cancer on health care systems by improving acceptance of AS and reducing overtreatment. Future studies with longer follow-up will need to determine whether HR-DWI can predict classic disease progression and help determine the optimal timing for surveillance imaging and biopsies when using HR-DWI. In this study, the sample size was too small and the study interval of 6–12 months between imaging was too short to assess whether imaging can predict true disease progression during AS.

It is well established that MRI can help detect clinically significant prostate cancer. A recent meta-analysis of randomized clinical trials representing 2,908 patients compared MRI-directed prostate biopsy with systematic biopsy, and use of MRI resulted in detection of 45% more clinically significant prostate cancers (22). However, there was no difference in the detection of clinically insignificant cancers, highlighting MRIs limitation in visualizing small, low-grade tumors, which are common in AS patients. Another important limitation of MRI is that some high-grade tumors are missed on MRI, especially when they are small. Vargas and colleagues compared MRI and prostatectomy, and when tumors with Gleason ≥4 + 3 were <0.5cc, only 26% of peripheral zone tumors and 20% of transition zone tumors were detected on MRI (23). In another study comparing MRI and prostatectomy, 76% of Gleason score 6 tumors and 30% of Gleason score 7 tumors were missed on MRI (24). These studies clearly point to need for better imaging technology.

During AS, cancer progression is monitored using serial biopsies because serial MRIs are unable to accurately predict cancer progression (25), despite attempts to standardize serial MRI reporting on AS (26). A recent retrospective study compared changes in prostate cancer volume on serial MRI during AS (7). The changes in tumor size for Gleason 3 + 3 prostate cancer and Gleason 3 + 4 prostate cancer were not significantly different, and the small increase in annual tumor growth was thought to be less than the interscan variability of serial MRIs. These findings are consistent with our observation that S-MRI has only a modest ability to predict Gleason 3 + 4 prostate cancer. Furthermore, several authors have documented that S-MRI can at times overestimate tumor volume (27), possibly due to adjacent inflammation and atrophy, and at other times underestimate tumor volume by over 50% (28).

Our HR-DWI technique improved the spatial resolution of S-DWI by approximately 3-fold using a combination of techniques. By decreasing the field-of-view, the resolution of the prostate was increased while peripheral signals that can produce artifacts and decrease resolution were suppressed (11, 16). During imaging, patient movement and rectal peristalsis can decrease resolution. Therefore, a motion compensation scheme based on gradient waveform designed to null movement was used to reduce the signal loss during the diffusion encoding step (12). It is important to note that it is possible to further increase resolution using these and other techniques (9). However, the increased resolution comes at the cost of increased scan time. Therefore, we settled on a 3-fold increase in resolution, to balance the need to improve resolution with need to maintain a clinically acceptable scan time. In future studies, our technology may be integrated with other technologies in development such as high-resolution DCE (29, 30), quantitative T1 and T2 mapping (31), super-resolution reconstruction (32), and artificial intelligence–driven image analysis (33).

The primary strength of this study is that it employs a novel HR-MRI to address an important problem in prostate cancer management. The prospective design, with S-MRI in same-patient controls that were acquired in the same setting, adds robustness to the findings. The MRI-ultrasound fusion biopsies, performed using lesions from both S-MRI and HR-MRI, ensured targeting of all suspicious lesions, and the addition of systematic cores ensured histologic characterization that is consistent with standard clinical practice. The radiologists and pathologists were blind to each other's findings, reducing the risk of interpretation bias. The S-MRI and HR-MRI were read in batches to decrease the risk of one image type influencing the reading of the other image type in the same patient. All images were read by two expert radiologists.

Our study has some limitations. First, the number of patients in our study was relatively small. However, the consistency of the results and the high statistical significance suggest that our findings are robust. Second, the reference standard was prostate biopsies, which is subject to sampling error. However, this was necessary when investigating a low-risk, small-volume cancer that is managed nonoperatively. Third, our study was performed at a single center and the imaging protocol was developed by our team. It remains to be seen whether the protocol can be implemented in other centers and whether the results can be replicated by non-GU radiologists. However, our single institution study is needed to make the case that other centers should investigate HR-DWI and commit to the necessary cost and slightly longer MRI scan times. Finally, future studies will need to determine whether use of HR-DWI can predict classic disease progression (e.g., progression from Gleason 6–7 to Gleason 8–10) and improve long-term clinical outcomes in patients with prostate cancer.

In conclusion, HR-DWI is a promising imaging technique that significantly improved the prediction of AH for men on AS for prostate cancer. This technique has the potential to reduce the clinical burden of AS by reducing the reliance on serial prostate biopsies. Future studies can validate our findings and determine whether HR-DWI can predict disease progression, and explore the utility of HR-DWI in other clinical settings such as prostate cancer diagnosis, staging, and mapping.

## Supplementary Material

Supplementary Tables S1, S2 S1: MR Protocol Parameters S2: Representativeness of Study Participants
