# Supplementary material for: High-resolution Diffusion-weighted Imaging to Detect Changes in Tumor Size and ADC, and Predict Adverse Biopsy Histology during Prostate Cancer Active Surveillance
Source: Cancer Res Commun. 2024 Mar 27;4(3):938–45. doi: 10.1158/2767-9764.CRC-24-0009 (PMC10967695; doi:10.1158/2767-9764.CRC-24-0009)
Supplement: Supplementary Tables S1, S2 — S1: MR Protocol Parameters S2: Representativeness of Study Participants [file crc-24-0009-s01.pptx]

## Slide 1
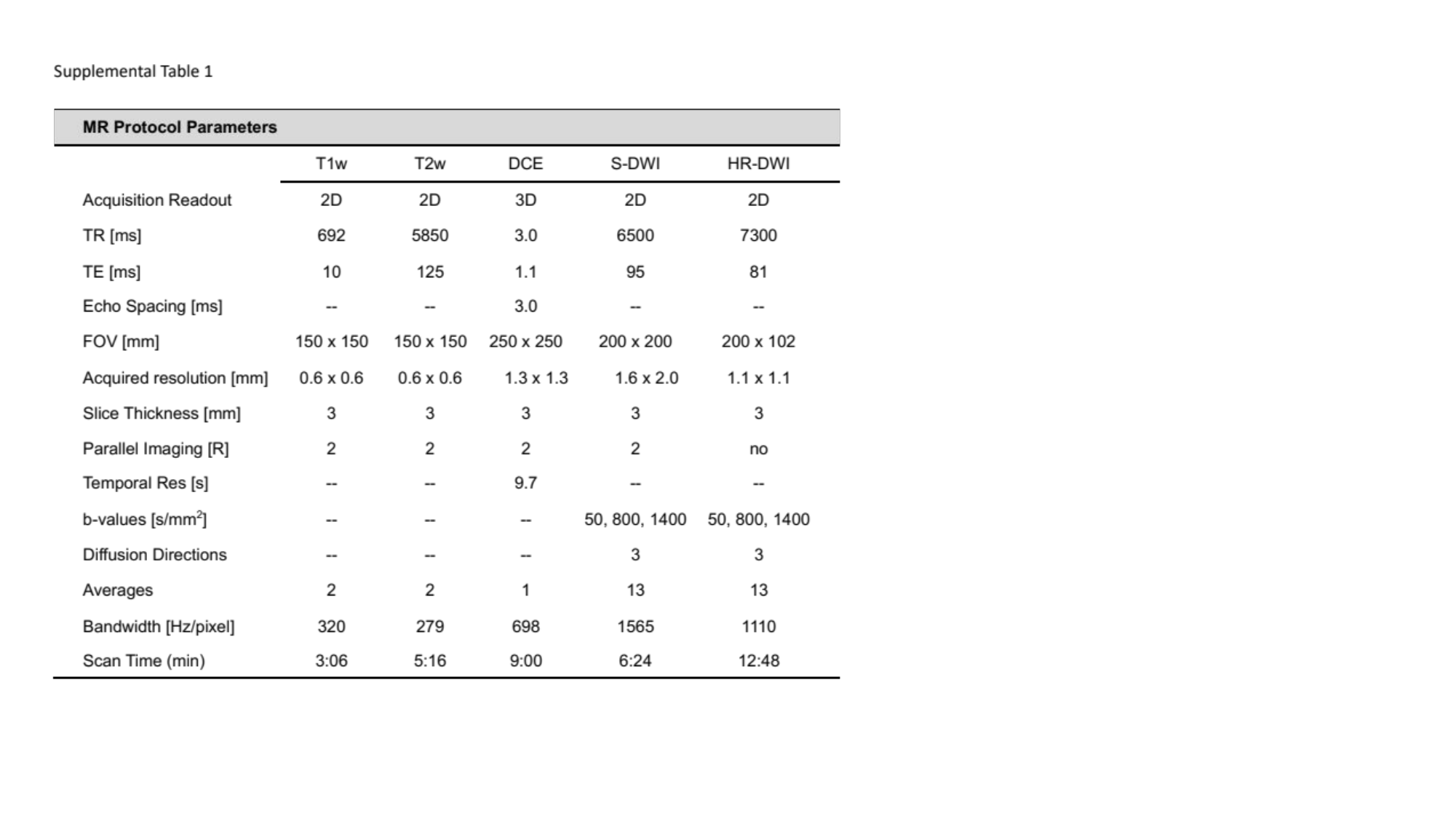

## Slide 2
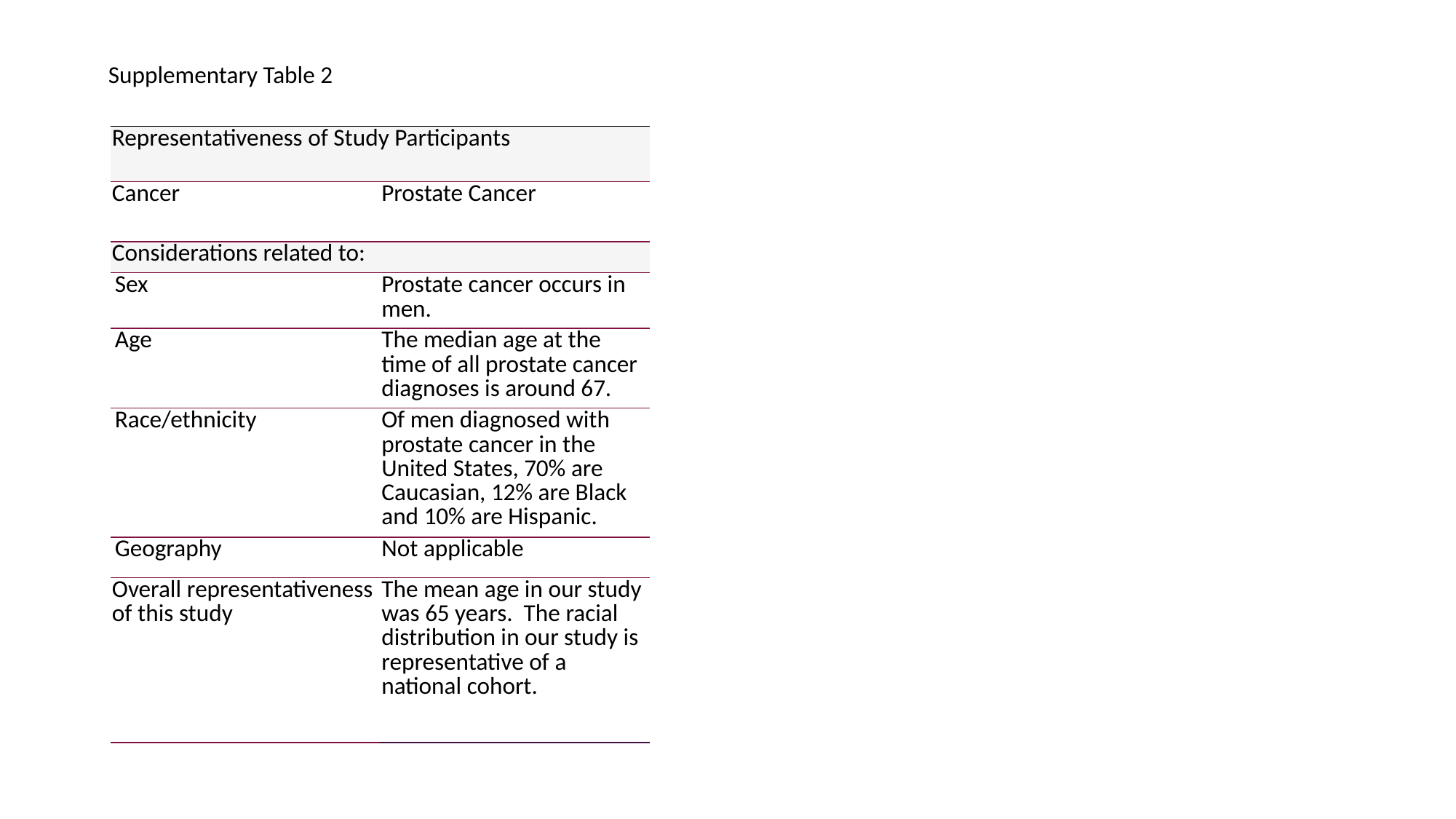

Supplementary Table 2
| Representativeness of Study Participants | |
| --- | --- |
| Cancer | Prostate Cancer |
| Considerations related to: | |
| Sex | Prostate cancer occurs in men. |
| Age | The median age at the time of all prostate cancer diagnoses is around 67. |
| Race/ethnicity | Of men diagnosed with prostate cancer in the United States, 70% are Caucasian, 12% are Black and 10% are Hispanic. |
| Geography | Not applicable |
| Overall representativeness of this study | The mean age in our study was 65 years. The racial distribution in our study is representative of a national cohort. |
